# Neoadjuvant intermediate-course versus long-course chemoradiotherapy in T3-4/N0+ rectal cancer: Istanbul R-02 phase II randomized study

**DOI:** 10.32604/or.2023.030351

**Published:** 2023-07-21

**Authors:** SUKRAN SENYUREK, SEZER SAGLAM, ESRA KAYTAN SAGLAM, HAKAN YANAR, KAAN GOK, DIDEM TASTEKIN, CANAN KOKSAL AKBAS, NERGIZ DAGOGLU SAKIN, GULBIZ DAGOGLU KARTAL, EMRE BALIK, METIN KESKIN, YASEMIN SANLI, MINE GULLUOGLU, ZULEYHA AKGUN

**Affiliations:** 1 Department of Radiation Oncology, Koc University School of Medicine, Istanbul, 34450, Türkiye; 2 Department of Medical Oncology, Demiroglu Bilim University Faculty of Medicine, Istanbul, 34394, Türkiye; 3 Department of Radiation Oncology, Istanbul University Oncology Institute, Istanbul, 34093, Türkiye; 4 Department of General Surgery, Istanbul University Istanbul Faculty of Medicine, Istanbul, 34093, Türkiye; 5 Department of Medical Oncology, Istanbul University Oncology Institute, Istanbul, 34093, Türkiye; 6 Department of Medical Physics, Istanbul University Oncology Institute, Istanbul, 34093, Türkiye; 7 Department of Radiology, Istanbul University Istanbul Faculty of Medicine, Istanbul, 34093, Türkiye; 8 Department of General Surgery, Koc University School of Medicine, Istanbul, 34450, Türkiye; 9 Department of Nuclear Medicine, Istanbul University Istanbul Faculty of Medicine, Istanbul, 34093, Türkiye; 10 Department of Pathology, Istanbul University Istanbul Faculty of Medicine, Istanbul, 34093, Türkiye; 11 Department of Radiation Oncology, Memorial Sisli Hospital, Istanbul, 34384, Türkiye

**Keywords:** Chemoradiotherapy, Rectal cancer, Neoadjuvant therapy

## Abstract

Radiation therapy (RT) is typically applied using one of two standard approaches for preoperative treatment of resectable locally advanced rectal cancer (LARC): short-course RT (SC-RT) alone or long-course RT (LC-RT) with concurrent fluorouracil (5-FU) chemotherapy. The Phase II single-arm KROG 11-02 study using intermediate-course (IC) (33 Gy (Gray)/10 fr (fraction) with concurrent capecitabine) preoperative chemoradiotherapy (CRT) demonstrated a pathologically complete response rate and a sphincter-sparing rate that were close to those of LC-CRT. The current trial aim to compare the pathological/oncological outcomes, toxicity, and quality of life results of LC-CRT and IC-CRT in cases of LARC. The prescribed dose was 33 Gy/10 fr for the IC-CRT group and 50.4 Gy/28 fr for the LC-CRT group. Concurrent chronomodulated capecitabine (Brunch regimen) 1650 mg/m^2^/daily chemotherapy treatment was applied in both groups. The European Organization for Research and Treatment of Cancer Quality of Life Questionnaire-Colorectal Cancer Module (EORTC QLQ-CR29) was administered at baseline and at three and six months after CRT. A total of 60 patients with LARC randomized to receive IC-CRT (n = 30) or LC-CRT (n = 30) were included in this phase II randomized trial. No significant difference was noted between groups in terms of pathological outcomes, including pathological response rates (ypT0N0-complete response: 23.3% *vs.* 16.7%, respectively, and ypT0-2N0-downstaging: 50% for each; *p* = 0.809) and Dworak score-based pathological tumor regression grade (Grade 4-complete response: 23.3 *vs.* 16.7%, *p* = 0.839). The 5-year overall survival (73.3 *vs.* 86.7%, *p* = 0.173) rate was also similar. The acute radiation dermatitis (*p* < 0.001) and any hematological toxicity *(p* = 0.004) rates were significantly higher in the LC-CRT group, while no significant difference was noted between treatment groups in terms of baseline, third month, and sixth month EORTC QLQ-CR29 scores.

## Introduction

The standard approaches to the use of radiation therapy (RT) in the preoperative treatment of resectable rectal cancer were short-course radiotherapy (SC-RT) alone or long-course chemoradiotherapy (LC-CRT) until recent years, although current treatment approach is to apply RT within total neoadjuvant therapy protocol [1–3]. It was reported in the randomized Polish II trial [4] that consolidation chemotherapy applied after SC-RT was not superior to LC-CRT, and three randomized trials that included stage T3-T4/N0+ locally advanced rectal cancer (LARC) patients indicated that LC-CRT provided promising results [5–7]. Although, quality of life assessment (QoL) is similar for both approaches, the most important differences are too more pathological complete response, negative circumferential margins, and sphincter preservation rate in LC-CRT [1,2]. Accordingly, preoperative LC-CRT has become a preferred treatment option in the management of LARC.

At present, data on the likelihood of using an intermediate-course of CRT (IC-CRT) without compromising the established effectiveness of LC-CRT in LARC patients are scarce [8]. The basis of this scheme is that complete the treatment before accelerated tumor repopulation begins and reduce chemotherapy toxicity by using capecitabine (an oral fluoropyrimidine) instead of bolus or continuous infusion 5-fluorouracil.

To the best of our knowledge, the only study to date to examine the utility of IC-CRT in T3-4/N0+ rectal cancer patients is a phase II single-arm study from Korea (KROG 11-02), which used an IC regimen of 33 Gy (Gray)/10 fr (fraction) with concurrent capecitabine [8]. The authors reported a pathologically complete response (ypCR) rate (13.8%) and a sphincter protection rate (91.3%) close to those achieved with LC-CRT. Although the positive circumferential resection margin (CRM) rate was also encouraging (6.25%), no data were available on long-term local recurrence and survival rates [8].

The present phase II randomized trial is the first known investigation in the literature to compare the efficacy of LC-CRT and IC-CRT in the management of LARC in terms of pathological and oncological outcomes, toxicity, and quality of life (QoL) status.

## Materials and Methods

### Study population

Patients with histologically proven infraperitoneal adenocarcinoma of the rectum; clinical T3/T4 or node negative/positive status on pelvic magnetic resonance imaging (MRI); no evidence of distant metastasis or secondary malignancy on positron emission tomography and computed tomography (PET-CT) scan at baseline; adequate bone marrow, liver, and renal function (leucocytes >4000/mm^3^, hemoglobin >10 g/dL, platelets >100000/mm^3^, serum bilirubin <1.5 mg/dL, serum transaminase <2.5 times the upper normal limit, serum creatinine <1.5 mg/dL); Karnofsky performance score ≥70; and those without uncontrolled diabetes, inflammatory bowel disease, pregnancy, infection, or cardiac contraindications for chemotherapy were included in the present phase II randomized study.

Patients were randomized to treatment arms after an initial assessment of a hemogram, marker levels (CEA, Ca 19-9), blood biochemistry, and a pelvic MRI and PET-CT scan in the treatment position. The block randomization (block size = 4) method was used to ensure a balance in sample size across groups over time.

Written informed consent was obtained from each patient following a detailed explanation of the objectives and protocol of the study. The protocol was registered to National Council of Higher Education Database (https://tez.yok.gov.tr/UlusalTezMerkezi/). The research was approved by the Istanbul University Faculty of Medicine Ethics Committee and conducted in accordance with the ethical principles stated in the Declaration of Helsinki.

### Assessments

Data reflecting patient demographic details (age, sex); tumor clinicopathological characteristics (location, clinical and pathological stage, lymphovascular invasion, perineural invasion); surgical treatment characteristics (surgical interval, surgery type, distal surgical margin, circumferential margin (CRM), number of harvested lymph nodes), adjuvant chemotherapy; treatment response (PET-response [T stage, N stage]); pathological outcome, including pathological response (complete response [ypT0N0], downstaging [ypT0-2N0]) and Dworak score-based pathological tumor regression grade (TRG); and oncological outcome (local recurrence, distant metastasis, 5-year overall survival [OS] and disease-free survival [DFS]) were recorded in the LC-CRT and IC-CRT groups.

### Study endpoints

The primary endpoint of this study was the pathological response rate in the LC-CRT *vs.* that of the IC-CRT group. The secondary endpoints were toxicity measurements and QoL assessments performed using the The European Organization for Research and Treatment of Cancer Quality of Life Questionnaire-Colorectal Cancer Module (EORTC QLQ-CR29) [9] in the two groups.

### Treatment

All of the patients underwent a CT simulation with a 5 mm slice thickness. The simulation was performed using a belly board apparatus and a full bladder protocol to reduce the toxicity to the small bowel and bladder. PET-CT images taken in the treatment position along with the CT simulation were combined for treatment planning.

The gross target volume (GTV) was determined according to the PET-CT fusion images. The clinical target volume (CTV) included the pelvic lymphatic area (at the L5/S1 interspace), all the mesorectum, and the GTV. For T3 disease, the pelvic lymphatic area was defined to comprise the obturator, internal iliac, and presacral lymph nodes. The external iliac lymph nodes were also included in cases of T4 disease. The planning target volume (PTV) was delineated as a 1 cm margin in all directions beyond the CTV. The prescribed dose was 33 Gy/10 fr for the IC-CRT group. The biologically effective dose (BED3 and BED10) values were similar to a standard SC radiation dose (25 Gy/5 fr) (Table 1).

**Table 1:** Comparison of biological equivalent doses of radiotherapy schemes

Regimen	BED Gy (α/β: 10 Gy) tumor control/acute complication probability	BED Gy (α/β: 3 Gy) late complication probability
LC-CRT 50.4 Gy/28 fr	59.47 Gy	80.64 Gy
IC-CRT 33 Gy/10 fr	43.89 Gy	69.30 Gy
SC-RT 25 Gy/5 fr	37.50 Gy	66.67 Gy

Abbreviations: BED: Biologically effective dose; IC-CRT: Intermediate course chemoradiotherapy; LC-CRT: Long-course chemoradiotherapy; SC-RT: Short-course radiotherapy.

In the LC-CRT group, 45 Gy/25 fr was prescribed and the PTV included a 1 cm margin in excess of the GTV, adding another 5.4 Gy/3 fr, for a total dose of 50.4 Gy/28 fr. Three-dimensional conformal RT and intensity-modulated RT were used to perform the planning. The patients were treated using a Siemens ONCOR (Siemens Healthineers, Erlangen, Germany) or a Truebeam STx linear accelerator system (TrueBeam STx; Varian Medical Systems, Palo Alto, CA, USA/Siemens Healthineers, Erlangen, Germany) and underwent daily digital portal imaging.

Concurrent chronomodulated capecitabine (Brunch regimen: morning and noon) 1650 mg/m^2^/daily was administered as chemotherapy in both groups [10]. Capecitabine was used in the IC-CRT arm for 10 working days. Adjuvant chemotherapy was left to the clinician’s decision according to the pathology results. Total mesorectal excision was performed after completion of the neoadjuvant therapy at between 8 and 12 weeks [11]. The patients underwent one of two surgical procedures according to the tumor location: abdominoperineal resection or low anterior resection.

### Pathological outcomes

The pathological TRG was defined using the Dworak score, which ranges from Grade 0 to Grade 4 [12]. In contrast to other classification systems, the highest Dworak score of Grade 4 (TRG 4) represents a complete response and indicates no viable cancer cells, while Grade 0 (TRG 0) signifies no regression or downgrading. The pCR was defined as the complete absence of a viable tumor and only fibrotic mass in the pathologic specimen. Acellular pools of residual mucin in specimens were considered to indicate a completely eradicated tumor. Reasons for electing to use the Dworak classification rather than the College of American Pathologists classification were described in detail in our previous study [13].

### Toxicity and quality of life assessment

Toxicity was evaluated according to the Common Terminology Criteria for Adverse Events v4.03 (CTCAE). QoL assessment was performed under the supervision of the physician by using the EORTC QLQ-CR29 [9]. All of the patients were asked to assess urinary frequency, urinary incontinence, dysuria, abdominal and anal pain, mucus and blood in the stool, dry mouth, sense of taste, body image, anal incontinence, stool frequency, sexual desire, erection problem, dyspareunia, and hair loss using a scale of 1 to 4 (1: Not at all, 2: A little, 3: Quite a bit, 4: Very much). The EORTC QLQ-CR29 was administered at baseline and again three and six months after CRT in patients who had not relapsed. The six-month EORTC QLQ-CR29 results were used to compare the level of chronic toxicity in the two treatment groups.

### Follow-up

Response assessment was performed six weeks after treatment and prior to surgery using PET-CT [14] and pelvic MRI scans. Follow-up of the patients was performed in three-month periods within the first two years and included a hemogram, blood biochemistry and tumor marker analysis, and pelvic MRI assessments.

### Statistical analysis

IBM SPSS Statistics for Windows, version 28.0 (IBM Corp., Armonk, NY, USA) was used to perform the statistical analysis. The descriptive data were expressed as mean, SD, minimum-maximum and percentage as appropriate. The ANOVA, independent sample *t*-test, Krusal-wallis and Mann-Whitney U test were used in the analysis of quantitative independent test. The chi-square (χ2) test and the Fisher exact test were used for the comparison of categorical data. Overall survival (OS) and disease-free survival (DFS) rates were analyzed based on the initiation of CRT. Sample size was determined with 5% Margin of Error, 80% Power and Standard Effect Size was determined as 0.75. According to that it was sufficient to include n = 28 cases in each group. The *p* < 0.05 was considered statistically significant.

## Results

### Baseline characteristics

A total of 67 patients with LARC randomized to receive IC-CRT (n = 35) or LC-CRT (n = 32) were included in this prospective phase II randomized trial conducted between March 2015 and September 2018. Two patients in the LC-CRT arm did not have surgery due to their comorbidities, two patients in the IC-CRT arm refused treatment before starting RT, and three patients did not come for follow-up after RT; therefore, those seven patients excluded from the analysis.

The two treatment groups were homogenous in terms of age and sex. The clinical and pathological characteristics of the tumor and treatment characteristics were also similar between the study groups. The median interval between surgery and neoadjuvant treatment was 12 weeks for all patients (10 weeks for the IC-CRT group and 13 weeks for the LC-CRT group). Sphincter-sparing surgery was performed on 86.7% of the patients in the LC-CRT group and 76.7% of the patients in the IC-CRT group. R0 resection performed on 90% of the patients in both the LC-CRT and IC-CRT groups (Table 2).

**Table 2:** Patient demographics, clinical/pathological stage characteristics and surgical details

	LC-CRT (n = 30)	IC-CRT (n = 30)	*p* value
**Patient demographics**			
Age, years; median, min-max	55 (32–76)	59 (41–80)	0.09
Sex, n (%)			
Female	10 (33.3)	15 (50)	0.190
Male	20 (66.7)	15 (50)	
**Clinical and pathological stage characteristics, n (%)**			
Clinical T stage, n (%)			
cT3	26 (86.7)	29 (96.7)	0.161
cT4	4 (13.3)	1 (3.3)	
Clinical N stage, n (%)			
cN negative	3 (10)	7 (23.3)	0.166
cN positive	27 (90)	23 (76.7)	
Radiological CRM, n (%)			
Positive	24 (80)	21 (70)	0.371
Negative	6 (20)	9 (30)	
Tumor location, n (%)			
Distal rectum	16 (53.3)	12 (40)	0.532
Middle rectum	14 (46.7)	18 (60)	
Pathologic tumor stage, n (%)			
ypT0	7 (23.3)	5 (16.6)	0.795
ypT1	2 (6.7)	0	
ypT2	8 (26.7)	11 (36.6)	
ypT3	11 (36.7)	14 (46.6)	
ypT4	2 (6.7)	0	
Pathologic nodal stage, n (%)			
ypN0	23 (76.7)	23 (76.7)	0.1
ypN1	5 (16.6)	4 (13.3)	
ypN2	2 (6.6)	3 (10)	
Lymphovascular invasion, n (%)			
Positive	2 (6.7)	3 (10)	0.640
Negative	28 (93.3)	27 (90)	
Perineural invasion, n (%)			
Positive	1 (3.3)	3 (10)	0.301
Negative	29 (96.7)	27 (90)	
Distal surgical margin, n (%)			
Positive	2 (6.7)	0 (0)	0.492
Negative	28 (93.3)	30 (100)	
CRM, n (%)			
Positive	2 (6.7)	3 (10)	0.640
Negative	28 (93.3)	27 (90)	
**Treatment characteristics, n (%)**			
Surgical interval			
8--12 weeks	19 (63.3)	20 (66.7)	0.787
>12 weeks	11 (36.7)	10 (33.3)	
Surgery type			
LAR	26 (86,7)	23 (76.7)	0.317
APR	4 (13,3)	7 (23.3)	
Number of harvested lymph nodes, median, min-max	15 (5–59)	14 (4–26)	0.261
Adjuvant chemotherapy, n (%)			
Yes	20 (66.6)	20 (66.6)	1
No	10 (33.3)	10 (33.3)	

Abbreviations: APR: Abdominoperineal resection; CRM: Circumferential resection margin; IC-CRT: Intermediate-course chemoradiotherapy; LAR: Low anterior resection; LC-CRT: Long-course chemoradiotherapy; Min-max: Minimum-maximum.

### Pathological and oncological outcomes

No significant difference was noted between the LC-CRT and IC-CRT groups in terms of pathological outcomes, including pathological response rate (ypT0N0-complete response: 23.3% *vs.* 16.7%, respectively; *p* = 0.519 and ypT0-2N0-downstaging: 50% for each; *p* = 0.809) or the Dworak score-based pathological TRG (Grade 4-complete response: 23.3% *vs.* 16.7%, respectively; *p* = 0.839) (Table 3).

**Table 3:** Treatment PET response, pathological response and oncological outcomes

	LC-CRT (n = 30)	IC-CRT (n = 30)	*p* value
**Treatment response, n (%)**			
PET response (T stage)			
Complete response	7 (23.3)	5 (16.7)	0.519
Partial response	12 (40)	12 (40)	
Stable	9 (30)	13 (43.3)	
Progression	2 (6.7)	–	
PET response (N stage)			
Complete response	16 (53.3)	10 (33.3)	0.188
Partial response	8 (26.7)	12 (40)	
Stable	3 (10)	1 (3.3)	
No lymph node	3 (10)	7 (23.3)	
**Pathological outcome, n (%)**			
Pathological response rate			
Complete response (ypT0N0)	7 (23.3)	5 (16.7)	0.519
Downstaging (ypT0-2N0)	15 (50)	15 (50)	
Dworak score (TRG)			
TRG 0	0	0	0.839
TRG 1	4 (13.3)	3 (10)	
TRG 2	14 (46.7)	15 (50)	
TRG 3	5 (16.7)	7 (23.3)	
TRG 4 (Complete response)	7 (23.3)	5 (16.7)	
**Oncological outcome**			
Local recurrence, n (%)			
Yes	3 (10)	0 (0)	0.237
No	27 (90)	30 (100)	
Distant metastasis, n (%)			
Yes	8 (26.7)	7 (23.3)	0.766
No	22 (73.3)	23 (76.7)	
5-year overall survival, %	73.3	86.7	0.107

Abbreviations: IC-CRT: Intermediate-course chemoradiotherapy; LC-CRT: Long-course chemoradiotherapy; PET: Positron emission tomography; TRG: Tumor regression grade.

**Figure 1:**
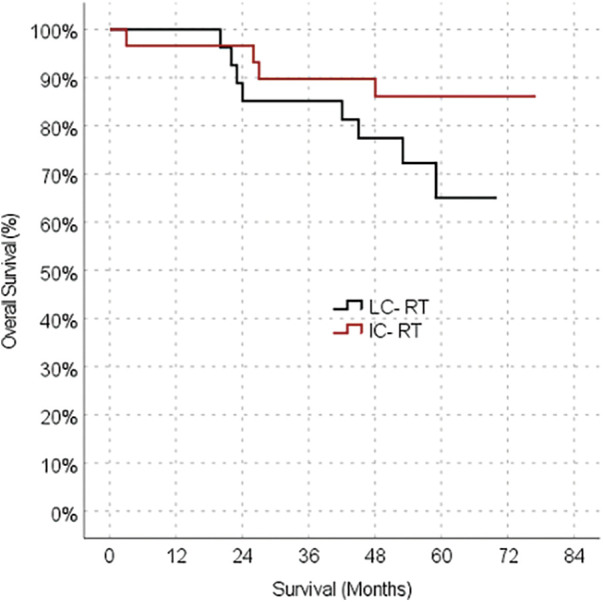
Kaplan-meier survival estimates for overall survival.

The median follow-up duration was 50 months (3–77 months) in the IC-CRT group and 52.5 months (4-70 months) in the LC-CRT group. The oncological outcome parameters were also similar between the LC-CRT and IC-CRT groups, including rates of local recurrence (10.0% *vs.* 0.0%, respectively; *p* = 0.237), distant metastasis (26.7% *vs.* 23.3%, respectively; *p* = 0.766), 5-year OS (73.3% *vs.* 86.7%, respectively; *p* = 0.173) (Table 2, Fig. 1).

### Toxicity and quality of life outcomes

The rates of acute radiation dermatitis (*p* < 0.001) and hematological toxicity (*p* = 0.004) were significantly higher in the LC-CRT group than in the IC-CRT group, while no significant difference was noted between the treatment groups in terms of acute gastrointestinal and genitourinary toxicity (Table 4).

**Table 4:** Acute toxicity data results

	LC-CRT (n)	IC-CRT (n)	*p* value
**Gastrointestinal toxicity, n (%)**			
Grade 0	5 (16.7)	3 (10)	0.747
Grade 1	16 (53.3)	17 (56.7)	
Grade 2	9 (30)	10 (33.3)	
Grade 3	–	–	
**Genitourinary toxicity, n (%)**			
Grade 0	4 (13.3)	3 (10)	0.728
Grade 1	17 (56.7)	20 (66.7)	
Grade 2	9 (30)	7 (23.3)	
Grade 3	–	–	
**Dermatological toxicity, n (%)**			
Grade 0	0	8 (26.7)	**<0.001**
Grade 1	15(50)	21 (70)	
Grade 2	15(50)	1 (3.3)	
Grade 3	–	–	
**Hematological toxicity, n (%)**			
Grade 0	17 (56.7)	27 (90)	**0.004**
Grade 1	12 (40)	2 (6.7)	
Grade 2	1 (3.3)	1 (3.3)	
Grade 3	–	–	

Abbreviations: IC-CRT: Intermediate-course chemoradiotherapy; LC-CRT: Long-course chemoradiotherapy.

Baseline, third month, and sixth month EORTC QLQ-CR29 scores revealed no significant difference between the LC-CRT and IC-CRT groups. Although there was deterioration in the QoL (total score) in the third month compared with the baseline level, the scores returned to the baseline level in the sixth month evaluation (Table 5).

**Table 5:** Quality of life total score comparison analysis results

	IC-CRT Med. ± SD	LC-CRT Med. ± SD	*p* value (m)
Total score			
Baseline	36.3 ± 4.2	36.0 ± 4.5	0.900
3 Month	41.8 ± 10.1	42.2 ± 7.7	0.539
6 Month	36.9 ± 10.1	39.6 ± 7.7	0.296
Difference from baseline			
Baseline/3 Month	5.5 ± 10.0	6.1 ± 6.8	0.399
	***p* = 0.004** *(w)*	***p* = 0.000** *(w)*	
Baseline/6 Month	0.6 ± 10.2	3.5 ± 7.8	0.524
	*p* = 0.349 (w)	*p* = 0.073 (w)	

Abbreviations: m: Mann-Whitney U test, w: Wilcoxon test.

However, analysis each question in both groups, an increase in urinary frequency, urinary incontinence, dysuria, erectile dysfunction, dyspareunia, interested in sex, frequent bowel movements during the night, sore skin around anal area, unintentional release of gas, leakage of stools, were noted a significant tendency for not to return the baseline levels. When analysis of difference to tendency for not to the return the baseline between groups; the frequent bowel movements during the night and sore skin around anal area was seen more in LC-CRT (*p*: 0.02 and *p*: 0.042, respectively).

## Discussion

To the authors’ knowledge, the current Istanbul R-02 Phase II study is the first prospective randomized study to compare the outcomes of IC-CRT and LC-CRT with a chronomodulated capecitabine (Brunch regimen) in LARC patients. The promising long-term data on the effectiveness of IC-CRT in LARC suggest that IC-CRT is associated with a similar pathological response and sphincter preservation rate to LC-CRT, as well as a better toxicity profile.

The encouraging results in sphincter preservation and R0 surgery in the current study seem to indicate that the efficacy of IC-RT is equivalent to that of long-term RT and allows for a shorter course of RT with concomitant use of chronomodulated capecitabine (Brunch regimen). A reduced dose of capecitabine (64%) could decrease toxicity.

The KROG 11-02 trial was a single-arm study conducted to evaluate a short CRT treatment protocol designed to prevent tumor repopulation by completing the therapy within two weeks and to increase the sensitivity of the tumor to radiation by using concurrent capecitabine. Surgery delayed in order to maximize the pathological response [8].

Our previous phase II brunch regimen study was based on the use of chronomodulated (morning and noon) capecitabine according to a specific time schedule as a part of neoadjuvant chemoradiation therapy in patients with LARC. Complete tumor regression was detected in 20% of the patients and no instance of grade 4 toxicity was seen [10].

In the current study, the pCR was 24% in the LC-CRT group and 17% in the IC-CRT group. Downstaging (ypT0-2N0) was detected in 50% of the patients in both groups. The RT scheme in the current study was similar to that used in the KROG 11-02 study and, the pCR rate was 13.8% (T0N0) and 33.8% had decreased stage (ypT0-2N0). The achievement of a better pathological response rate might be associated with the use of the brunch capecitabine regimen.

In the KROG 11-02 study, the median interval until surgery was reported to be 7.5 weeks [8]. In our study, all of the patients in both groups underwent surgery at a median of 8 to 12 weeks after the CRT; this duration corresponds to delayed surgery in the literature, which is associated with an increase in pathological response for all radiation scheme [11,15–18].

Sphincter-sparing surgery and R0 resection are important success parameters of a neoadjuvant protocol. In our study, high rates of sphincter preservation and R0 resection close to those of the KROG-11 trial were obtained in both groups (LC-CRT: 87% and 90%, respectively, and IC-CRT: 77% and 90%, respectively) in infraperitoneal tumors. Three local recurrences were observed in the LC-CRT group among patients who had positive surgical margins; the recurrence ratio was 10% for LC-CRT patients and 5% for all of the patients. The numerically higher N+, distal tumors, and greater radiological CRM involvement may have increased the likelihood of local recurrence in LC-CRT group. Several prospective studies and the Istanbul R-01 study have demonstrated similar local recurrence rates. Further subgroup analyses revealed local recurrence to be significantly more common in patients with distal tumors (0–5 cm) than those with proximal tumors (16.2% *vs.* 6.3%, respectively; *p* = 0.045) [1,2,7,19,20].

In the current study, the baseline, third month, and sixth month-CR29 scores revealed no significant difference between groups. Although erectile dysfunction was reported significantly more frequently in the IC-CRT arm in our first series, published in American Society for Therapeutic Radiation Oncology 2018 [21], the significance was not maintained in the current series with a larger number of patients. Most studies that have examined toxicity and QoL have evaluated the difference between short-term and long-term treatment modalities. Comparable rates for late toxicity were reported, regardless of the duration of treatment, whereas lower rates for acute toxicity were seen in patients who underwent SC treatment [22]. Guckenberger et al. [23] used both the EORTC QLQ-C30 and -CR29 questionnaires in their study comparing LC-CRT and SC-RT and found that only the physical activity item score was better in the LC-CRT arm. In the another recent study that analyzed long term patient reported bowel toxicity and QoL were found similar in both LC-CRT and SC-RT [24].

As a result of advancing knowledge in all of these areas of discussion, the treatment approach in rectal cancer has been shifting to a total neoadjuvant protocol and an organ-preserving approach in recent years [25–28]. Both SC and LC-CRT modalities continue to be examined and assessed. IC-CRT is a new modality; however, the encouraging first data merit additional research and suggest that it may find a place among total neoadjuvant protocols.

The primary limitation of our study is the small sample size. A larger number of cases and additional substantiating data will undoubtedly increase physicians’ confidence in IC-CRT.

## Conclusion

Our study results indicated that IC-CRT therapy could be a suitable substitute for LC-CRT therapy due to the absence of any significant difference in the rates of pathological response and sphincter-sparing surgery, as well as offering the advantage of less risk of acute any hematological and skin side effects as a result of radiation. A Phase III randomized non-inferiority study would provide valuable additional information.

## Data Availability

The data that support the findings of this study are available from the corresponding author, Sezer Saglam (saglam@istanbul.edu.tr), upon reasonable request.
